# Intentions on contraception use and its associated factors among postpartum women in Aksum town, Tigray region, northern Ethiopia: a community-based cross- sectional study

**DOI:** 10.1186/s12978-018-0632-2

**Published:** 2018-11-09

**Authors:** Teklehaymanot Huluf Abraha, Hailay Siyum Belay, Getachew Mebrahtu Welay

**Affiliations:** 1 Department of Reproductive Health, School of Public Health, College of Health Sciences, Aksum University, P.O. Box: 298, Aksum, Tigray Ethiopia; 2 Department of Epidemiology and Biostatistics, School of Public Health, College of Health Sciences, Aksum University, Aksum, Tigray Ethiopia

**Keywords:** Contraceptive use, Intention, Postpartum period, Factors, Aksum, Tigray, Ethiopia

## Abstract

**Background:**

Increased access to contraceptive methods has been established as a cost-effective strategy for developing countries to reduce maternal and child mortality. Intentions to contraceptive uptake appear to be best predictors of actual contraceptive practice than the unmet need. However, intention to contraceptive use in Ethiopia particularly among postpartum women is not well assessed. Therefore, the objective of this study was to assess intention to use modern contraceptive and to identify factors associated among postpartum women in Aksum town.

**Methods:**

A community –based analytical cross-sectional study design was done to collect the data from 604 postpartum mothers using a structured questionnaire. The data was collected from March 25 to April 24, 2015. A multivariable logistic regression was conducted to assess factors associated with intentions to use contraceptive methods. Factors influencing intentions on contraceptive methods use were assessed by computing adjusted odds ratios (AOR) at 95% confidence interval (CI) with statistical significant *p*- value < 0.05.

**Results:**

Intention to use modern contraceptive was 84.3%. Resumed sexual intercourse (AOR = 1.78; 95% CI: 1.34, 3.92) and women whose their husband approved family planning to use (AOR = 1.57; 95% CI: 2.02, 5.57) were more likely to have intention on contraceptive use. In addition, those women who knew at least one method of modern contraceptive (AOR = 5.17; 95% CI: 1.69, 15.82) were more likely to had intention to use modern contraceptive during extended postpartum period compared to their counterparts.

**Conclusion and recommendation:**

More than eight in ten study participants have intention to use contraceptive in the Aksum town. Resumed sexual intercourse, husband’s approval of family planning and knew at least one method of contraceptive are the three major predictors to be an areas when considering interventions to increase of intention on contraceptive. Therefore, this study highlighted that; in order to increase intention and adoption of contraceptive, the family planning services providers and programmers should continue the promotion of partner involvement and increasing family planning knowledge through printed media and mass media.

**Electronic supplementary material:**

The online version of this article (10.1186/s12978-018-0632-2) contains supplementary material, which is available to authorized users.

## Plain English summary

### Background plain

Family planning is an integral component of Maternal and Child Health (MCH) services in the Ethiopia health system. However, unmet need of contraceptive during Extended Postpartum Period in Ethiopia is unacceptable high. This exposes the mothers to high risk of unwanted pregnancies and unsafe abortion. This study is the first to assess the level of intention on contraception among postpartum women in Ethiopia, particularly in the study area. Measuring the prevalence of intention for contraception in the extended postpartum period is pivotal for designing and evaluating postpartum family planning programs. Finding from this study could help family planning planners and services providers to have a good insight of predictors of intention on contraceptive adoption and to take necessary interventions.

## Methods

Between March 25 to April 24, 2015, was conducted a study among 604 postpartum women in Aksum town, Tigray region, northern Ethiopia. We conducted analytical cross- sectional study design using a systematic random sampling technique. A structured item questionnaire was used to collect data on the socio-demographic, knowledge of mother regarding family planning, maternal and reproductive health related variables. Data cleaning and analysis was carried out with the aid of IBM Statistical Package for the Social Sciences (SPSS) version 20 for Windows. Descriptive analysis was used to explore the characteristics of the study participants.

## Results

Majority of study participants intended to use contraceptive during the Extended Postpartum Period (EPP). Women who resumed sexual intercourse, husband approved family planning to adopt and know at least one method of contraceptive were identified factors associated with intention to use contraceptive.

## Conclusion and recommendation

Despite the fact that majority of postpartum women was intended to use, but the level of contraceptive adoption found low (48%). Therefore, in order to increase intention and adoption of contraceptive husband/partner involvement is mandatory.

## Background

Contraceptive utilization is a key predictor in preventing unwanted pregnancies, reducing maternal and child mortality, and improving the lives of women and their families [1, 2]. In addition, it is critical in achieving towards the 2030 Sustainable Development Goals (SDGs) [3]. Intention to use postpartum modern contraceptive methods is a better measurement for contraceptives in the extended postpartum period than the unmet need. In addition, by expressing the intention to use contraception, women are able to better visualize their future need and are more likely to translate it in to actual practice [4, 5].

Extended Postpartum Period (EPP) provides a window of opportunity for women on the adoption of modern contraceptive methods [6–8]. Despite the fact that, in developing countries many postpartum women fail to obtain family planning services soon after birth and become pregnant again, either much sooner than they wish or contrary to their desire to cease child bearing entirely [5].

A study conducted in 21 low and middle income countries found that 90% of postpartum women intended to adopt contraceptive in future [5]. In United State of America (USA), the proportion of women who expressed an intention to use modern contraceptive methods was estimated 91% [9]. Research evidence have showed that, maternal age, antenatal care service use, prior use of any contraceptive use [10], knew at least one contraceptive method [11] had been identified as factors associated for intention to use contraceptive.

About 86% of women in Ethiopia have an unmet need for family planning methods in the Extended Postpartum Period (EPP) [6]. Each year, 1.9 million Ethiopian women had an intended pregnancy. In Tigray, contraceptive utilization among postpartum women is low [12, 13]. Federal Ministry of Health set an objective to increase family planning adoption from 42 to 55% by 2020 [14]. Among the general population, the factors affecting women’s intentions to adopt contraception methods had been studied extensively [11, 15–18]. However, intention on contraceptive use in Ethiopia among postpartum women is a great limitation of the literature. Therefore, the objective of this study was to file these gaps and to assess intention to use modern contraceptive and identifying determinant factors among postpartum women in Aksum town. The finding from this research project could help planners, programmers, nongovernmental organizations (NGOs) and decision makers to have a good insight of factors intention on contraceptive utilization and to take appropriate interventions and may result in steeper increase in the uptake of family planning methods and a reduction in maternal mortality, child mortality, infant, neonatal mortality and unintended pregnancies rate.

## Methods

### Study design and study setting

A community-based cross-sectional study design was carried out between March 25 to April 24, 2015 in Aksum town, Tigray region. According to [19], the town administrative health office report the estimated total population 60,706, of whom 30,960 are women living in five *kebeles* (the smallest administrative unit). Majority of the population depends on none agriculture production. The town has achieved universal health coverage. There are 2 public Health Centers, 1 referral hospital (owned by Aksum University), 1 Zonal Hospital, 5 private clinics and 11 drug shops providing maternal, child and other health services to the population.

### Sources population

All reproductive age women who gave birth in the last 12 months prior to the study period (March 25, 2015-April 24, 2015) and who lived in Aksum town for more than 6 months were taken as sources population.

### Study population

All reproductive age women who gave birth in the last 12 months during the data collection period (March 25, 2015- April 24, 2015) in the randomly selected *kebeles*.

#### Inclusion criteria

The study target women who had gave birth in the last 12 months, regardless of parity, ethnicity, religion, income, education, maternal age, maternal health services, sexual activities, menses status, knowledge of contraception and use were included.

#### Exclusion criteria

Women who were unable to speak or not hearing, those who had a still birth and/or neonatal deaths with the previous pregnancy and hysterectomy.

### Sample size and sampling technique

A single population proportion formula was used to determine the sample size considering the following assumptions: Since there is no a study in Ethiopia, the proportion women who have intentions on contraception use methods in extended postpartum period was assumed to be 50, 95% CI, 5% absolute level of precision [20, 21], and 1.5% design effect. In addition a none response rate 5% was used and finally a sample size of 604 was determined. From the four *kebele* in Aksum town, two *kebele* were selected using a lottery method. The samples size was allocated by using proportional allocation to size (PAS) to the total number of postpartum women in the *kebeles.* A total of 604 study participants were selected by systematic random sampling technique (Additional file 1. Figure S1). Before the data collection; all eligible study participants who gave birth within the past 1 year in the randomly selected *kebeles* were traced using house-to-house survey.

### Operational definition and definition of terms

#### Intention to use a modern contraception

Intention to use a modern contraception refers to all women who were found in the extended postpartum period who are not currently using any modern contraception but reporting intent to use any of modern contraceptive at some time in the future. A binary dependent variable indicating intention use any method of the modern contraceptive coded as “1” and no intention coded as “0”.

#### Knowledge of modern contraceptives

Knowledge of modern contraceptives refers to the study participants’ spontaneously mentioned at least one of the modern contraceptives (pills, injectable, implants, IUD, female and male sterilization) [22].

#### Extended Postpartum Period (EPP)

The first-twelve month period after a live birth [7].

#### Birth interval

It is the length of time between two successive live births [23].

#### Parity

The number of children ever given birth.

#### Want space

Are those who are fecund (able to become pregnant) but want to postpone their next birth for 2 or more years [24].

#### Want to limit

Are those who are fecund (able to become pregnant) but are saying they do not want another child [24].

### Data collection

Data was collected via face to face interview at the study participant’s home using a structured and pre-tested questionnaire. The tool was prepared originally in English and translated to local language (*Tigrigna*) and translated back to English in order to maintain internal consistency. The tools was adopted from similar studies [9, 10], but customized for this study. The questionnaire included sections on socio demographic characteristics of respondents, their maternal health service use, their reproductive intentions, their knowledge and current adoption and their intentions to use contraceptive methods among postpartum women. Four female diplomas Midwifery and one BSc Nurse were participated in the data collection process. They had given 2 days training before deploying to actual work. The supervisors and the principal authors supervised the data collection process and checked the data completeness on daily bases.

### Statistical analysis

Data were entered and cleaned using EPI-Info version 7(Centers for DiseaseControl and Prevention, Atlanta, GA, USA) [25] and exported to SPSS version 20 (SPSS Inc., Chicago, IL, USA) for analysis. Descriptive statistics were done presented in the form of tables and texts. Both binary and multiple logistic regressions were carried out to identify associated factors. The explanatory variables (*p* < 0.2) were then further analyzed by logistic regression. Factors influencing to intentions on contraceptive methods use were assessed by computing adjusted odds ratios(AOR) at 95% confidence interval(CI) with statistical significant *p-* value < 0.05. The final model was assessed for multicolliniarity using Variance Inflation Factor(VIF) [26] and goodness of fit using Hosmer and Lemishow test [27].

## Results

### Socio-demographic variables

In this study, 604 postpartum women were interviewed. From these, 590(97.7%) women were responded to the questioner. The mean age of the study participants was 27.4 ± 5.0 years. Two hundred thirty one (39.2%) were aged between 25 and 29 years; 3.2% were teenagers (< 20 years). Three hundred eighty three (64.9%) were housewives. Almost all (99.3%) were Tigray by ethnicity, the rest were Amhara. Of the respondents, 546 (92.5%) were Orthodox Tewohado Christians, and the rest were Muslim. Three hundred eight (52.2%) of the study participants had attended secondary and above educational level and 38.5% of their partner attended primary educational school (Table 1).

**Table 1:** Socio- demographic variables in Aksum town, Tigray region, northern Ethiopia, June, 2015

Variables	Frequency(n)	Percentage (%)
Age		
16–19	19	3.2
20–24	149	25.2
25–29	231	39.2
30–34	126	21.4
35–49	65	11.0
Marital status		
Married	543	92.0
Others^a^	47	8.0
Educational status		
No formal education	80	13.6
Primary education	202	34.2
Secondary education and above	308	52.2
Partner’s education (*n* = 545)^b^		
No formal education	24	4.4
Primary school	210	38.5
Secondary school and above	311	57.1
Mother occupation		
House wife	383	64.9
Government employee	46	7.8
Private employee	103	17.5
Daily labourer	43	7.3
Others^c^	15	2.5
Partner occupation (*n* = 545)^b^		
Government employee	125	22.9
Private employee	257	47.2
Daily labourer	131	24.0
Others^d^	32	5.9
Monthly income (Ethiopian Birr)		
≤600	150	25.4
601–1000	159	26.9
1001–2000	154	26.1
≥200	17	21.5

^a^Single, separated, divorced, or widowed

^b^Among married women

^c^Alcohol /Swa seller, farmer, or student

^d^Farmer, pension, or guard

### Maternal and reproductive health services use

In this study a total 579 or 98.1% of the study participants attended at least one antenatal care (ANC) visits. Furthermore, 85 and 13.1% of the study participants received antenatal care from hospital and health centers respectively. Five hundred twenty four of the women had received four or more antenatal care visits. Five hundred seventy seven delivered by skill attendants at health institution (Health Center or Hospital). Two hundred fifty eight (43.7%) had received postnatal care (PNC) either at health center or hospital. Among all women 68.3% of them had resumed sexual intercourse at the time of survey. One hundred fifty nine (39.45%) of the study participants start sexual intercourse at 6 week of postpartum period. More than three forth (77.5%) of women got family planning counseling in their home by the town health extension workers (HEWs) in the last 12 months. Two hundred eighty three (48%) study participants were using modern contraceptive (Table 2).

**Table 2:** Characteristics of maternal and reproductive health services use in Aksum town, Tigray region, northern Ethiopia, June, 2015 (*n* = 590)

Variables	Frequency(n)	Percentage (%)
Parity (*n* = 590)		
1–4	536	90.8
> = 5	54	9.2
Currently living children (*n* = 590)		
1	182	30.9
2–3	281	47.6
> = 4	127	21.5
Birth interval (*n* = 416)		
< 24 months	105	25.2
24–47 months	131	31.5
> = 48 months	180	43.3
Antenatal care (*n* = 590)		
Yes	579	98.1
No	11	1.9
ANC visits (*n* = 581)		
1–3	57	9.4
> = 4	524	90.6
Place of birth (*n* = 590)		
Health institution	577	97.8
Home	13	2.2
Postpartum duration (*n* = 590)		
0–12 Weeks	186	41.5
13–26 Weeks	198	33.6
27–38 Weeks	141	23.9
39–52 Weeks	65	11.0
Postnatal care (*n* = 590)		
Yes	258	43.7
No	332	56.3
Who decide to use FP (*n* = 590)		
Mainly respondents	104	19.1
Mainly husband	36	6.6
Jointly decision	405	74.3
Postpartum menstruation status (*n* = 590)		
Menstruating	205	34.8
Amenorrhea	385	65.2
Resumed sexual intercourse (*n* = 590)		
Yes	403	68.3
No	187	31.7
Reproductive intention (*n* = 590)		
Want to have space	396	67.1
Want to limit	86	14.6
Undecided	99	16.8
Want to have a child soon	9	1.5

### Intentions on modern contraceptive use methods in the extended postpartum period

The prevalence of intention to use modern contraceptive was 84.3%. Of those who had intentions, 215(83.3%) and 43(16.7%) had intentions to adopt for purpose of spacing and limiting respectively. Nearly two third (66.4%) of the study participants used modern contraceptive prior to the last child. Regarding knowledge of modern contraceptive almost all 95.8% of the postpartum women were found that knowledgeable on the modern contraceptive methods. The least mentioned type of modern contraceptive use was male and female sterilization (Table 3).

**Table 3:** Knowledge and practice of modern contraceptives of postpartum women in Aksum town, Tigray region, northern Ethiopia, June, 2015 (*n* = 590)

Variables	Frequency (n)	Percentage (%)
Know at least one modern contraceptive		
Yes	564	95.6
No	26	4.4
Type of modern contraceptives known		
Injectable	512	86.8
Pills	407	69.0
Implants	406	68.8
IUD	285	48.3
Male condom	120	20.3
Female sterilization	44	7.5
Male sterilization	24	4.1
Heard contraceptive massage in the last 12 months(mass media, print media)		
Yes	564	95.6
No	26	4.4
Use of modern contraceptives		
Yes	283	48.0
No	307	52.0
Intend to use contraceptive methods(n=258^a^)		
Yes	258	84.3
No	49	15.7

^a^(Among currently not used contraceptive)

### Factors associated with intention to use modern contraceptive

The multivariable logistic regression model showed that, resumed sexual intercourse, husband family planning approval, knew at least one modern contraceptive were found significantly associated with the dependent variable.

Women who were resumed sexual intercourse at the time of survey (AOR = 1.78; 95% CI: 1.34, 3.92] had higher odds of intention on modern contraceptives compare to women who were not resumed sexual intercourse. There was significantly association of intention to utilize methods of contraceptive among women who their husband approved family planning to use (AOR = 1.57; 95% CI: 2.02, 5.57) compares to their counterparts. Those women who were know at least one method of modern contraceptive have 5.17 times higher odds to intention to use modern contraceptive during extended postpartum period compare to their counterparts (AOR = 5.17; 95% CI: 1.69,15.82) (Table 4).

**Table 4:** Factors associated with intention to use modern contraceptive methods in the future among postpartum women, in Aksum town, Tigray region, northern Ethiopia, June, 2015

Variables	Intention to use contraceptives		COR(95% CI)	AOR(95% CI)
	Yes (%)	No (%)		
Parity				
1–4	226(84.9)	40(15.1)	1.41(1.34,3.28)	2.31(0.82,2.93)
> = 5	32(80)	8(20)	1(ref)	1(ref)
Menses status				
Resumed	40(81.6)	9(18.4)	1.25(1.12, 2.79)	1.22(0.45,3.28)
Not resumed	218(84.8)	39(15.2)	1(ref)	1(ref)
Resumed sexual intercourse				
Yes	115(83.3)	23(16.7)	1.14(1.12, 2.12	1.78(1.34,3.92)
No	143(85.1)	25(14.9)	1(ref)	1(ref)
Ever discussed with husband regarding FP				
Yes	192(92.3)	16(7.7)	4.80(2.31,9.93)	2.27(0.62,8.31)
No	50(71.4)	20(28.6)	1(ref)	1(ref)
Husband FP approval				
Yes	183(92)	16(8)	3.87(1.88,7.96)	1.57(2.02,5.57)
No	59(74.7)	20(25..3)	1(ref)	1(ref)
Know at least one modern contraceptive				
Yes	248(87.3)	36(12.7)	8.26(3.33,20.51)	5.17(1.69,15.82)
No	10(45.5)	12(54.5)	1(ref)	1(ref)

Ref: Reference Category

## Discussion

In this study the prevalence of intention to use on modern contraceptive among postpartum women was 84.3%. This result was slightly lower than the finding in United State of America which reported [90%] [9]. This variation could be explained by the difference of study setting, accesses to information and availability to services use.

Two hundred fifteen (83.3%) and 16.7% of the postpartum women want to space and to limit respectively. Moreover, their intention on contraceptive methods was adequate. This tell us, the skilled attendants and family planning providers needs to evaluate the reproductive goal of the women during postpartum period and inform them the availability contraceptive methods during postnatal follow up and immunization period. This could be due to the current the government tireless working to improve the knowledge and practice of contraceptive using different strategies (i.e. availing different contraceptive with free of charge, community based contraceptive distribution and providing information using media outlets) and economic burden condition.

More than 95% of the study participants were mentioned at least one modern contraceptive. This result was in line a study conducted in Ethiopia. This finding might be explained by the fact that due to the efforts done by the Ethiopian government health policy and non government organizations (NGOs) to strengthen for maternal health and family health strategies like: the different community and institutional-based reproductive health services and health education being given by health workers, introducing health development army (HDA) and community health insurance have a positive effect for the increasing knowledge level of family planning [14].

The most well known modern contraceptive methods were injectable and pills. Among all postpartum women, the permanent contraceptives were least known (Table 3). This was in line the finding in Ethiopia [24, 28].

Women who were resumed sexual intercourse at the time of survey key predictors on intending to use methods of contraceptive. This may be due to when women resume sexual intercourse; they perceive themselves to be at high risk for pregnancy, which motivates them to use contraceptive methods in the future. Therefore, resuming sexual intercourse is strongly linked to the intention of contraceptive methods in the extended postpartum period (EPP) [5].

There was significantly associated with intention to use methods of contraceptive among women who were their husband approved family planning to use. This finding indicated that the importance of focusing on male involvement in family planning efforts because husband / partner do seem to play a key role in deciding future contraceptive methods for their wives [29, 30].

Study participants who knew at least one modern contraceptive method had more likely of intention to use contraceptive methods compared to those who did not knew any methods of contraceptive. This in line a study conducted in Adigrat town, Tigray region, Ethiopia [11].

### Limitations

This study used a cross-sectional study, which can only showed association rather than causal relationships between the different independent variables and the dependent variable. The findings from this study are internally and externally valid and provide a clear picture of the situation on intention on modern contraceptive use among postpartum women in Aksum town.

## Conclusion and recommendation

More than eight in ten study participants have intention to use contraceptive in the Aksum town. The factors affecting intention to use modern contraceptive method were resumed sexual intercourse; postpartum women who were their husband approved family planning to use and knew at least one type of modern contraceptive. Therefore, this study recommend that; in order to increase intention and use of contraceptive, the family planning services providers & programmers should continue the promotion of husband / partner involvement and family planning knowledge through printed media and mass media. In addition, the town administrative health office should design health education programs that promote contraceptive knowledge and practice during extended postpartum period.

## Additional file

Additional file 1: **Figure S1.** Schematic representation of sampling procedure, intention on contraceptive use and associated factors among postpartum women in Aksum town, Tigray region, northern Ethiopia, June 2015 (*n* = 604). (DOCX 17 kb)

## Abbreviations

AOR: Adjusted odds ratio
CI: Confidence interval
COR: Crude odds ratio
EPP: Extended postpartum period
FP: Family planning
HAD: Health development army
HEW: Health extension worker
IRB: Institutional review board
IUD: Intrauterine devices
MCH: Maternal and child health
MPH: Master of public health
NGOs: Non government organizations
PNC: Postnatal care
SDGs: Sustainable development goals
SPSS: Statistical package for the social sciences
VIF: Variance inflation factor
