# Supplementary material for: Intentions on contraception use and its associated factors among postpartum women in Aksum town, Tigray region, northern Ethiopia: a community-based cross- sectional study
Source: Reprod Health. 2018 Nov 9;15:188. doi: 10.1186/s12978-018-0632-2 (PMC6234798; doi:10.1186/s12978-018-0632-2)
Supplement: Supplementary file 1 — Figure S1. Schematic representation of sampling procedure, intention on contraceptive use and associated factors among postpartum women in Aksum town, Tigray region, northern Ethiopia, June 2015 (n = 604). (DOCX 17 kb) [file 12978_2018_632_MOESM1_ESM.docx]

The two k*ebele* were selected by lottery method

Proportional allocation to size of postpartum women to each  *kebele*

Figure 1
